# Supplementary material for: Temporal Evaluation of Insecticide Resistance in Populations of the Major Arboviral Vector Aedes Aegypti from Northern Nigeria
Source: Insects. 2022 Feb 10;13(2):187. doi: 10.3390/insects13020187 (PMC8876019; doi:10.3390/insects13020187)
Supplement: Supplementary file 1 [file insects-13-00187-s001.zip › Figure S1.pdf]

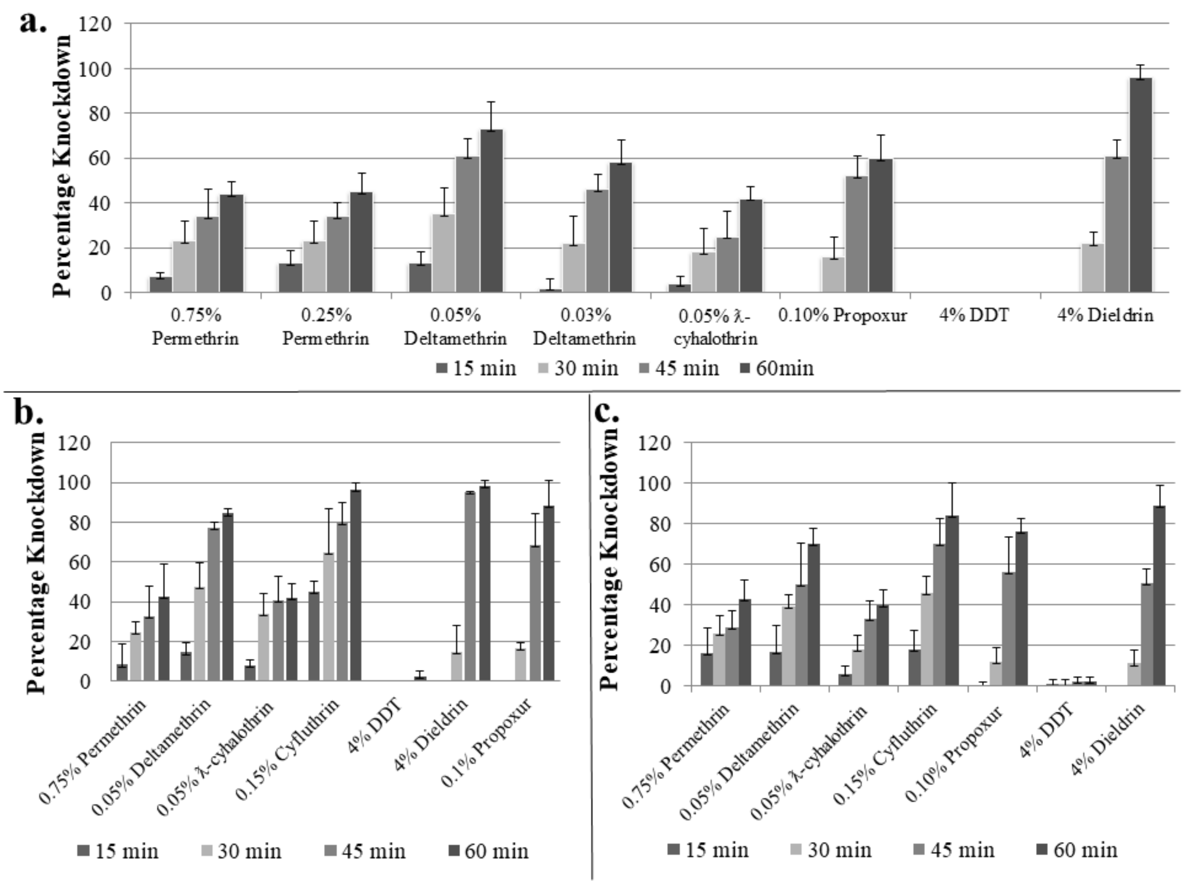

Figure S1. Results of WHO tubes bioassays with public health insecticides. Results are averages of percentage knockdown for 15, 30, 45 and 60 min, with error bars indicating standard deviations. (**a**–**c**) indicate results from 2020, 2019 and 2018, respectively for Kano populations.
