# Supplementary material for: Temporal Evaluation of Insecticide Resistance in Populations of the Major Arboviral Vector Aedes Aegypti from Northern Nigeria
Source: Insects. 2022 Feb 10;13(2):187. doi: 10.3390/insects13020187 (PMC8876019; doi:10.3390/insects13020187)
Supplement: Supplementary file 1 [file insects-13-00187-s001.zip › Figure S2.pdf]

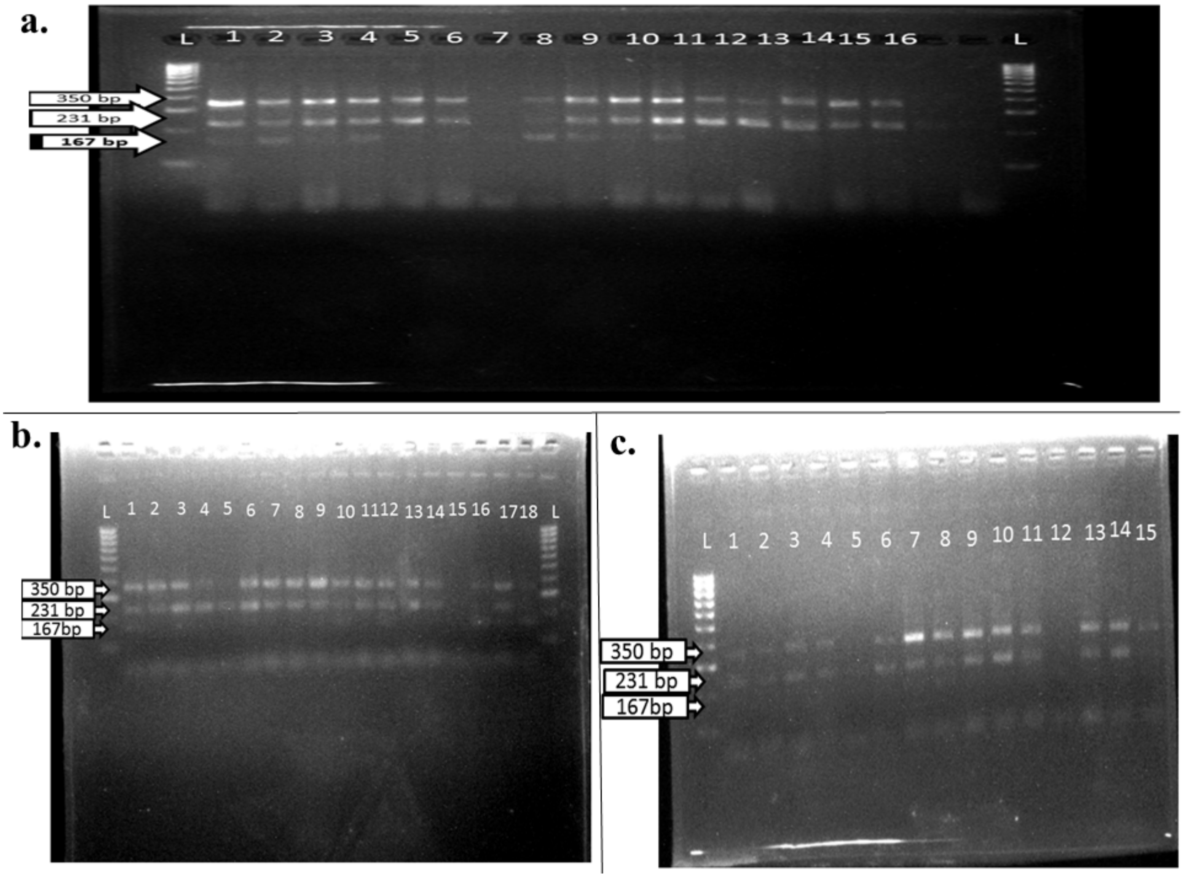

Figure S2. Representative agarose gel of allele specific PCR genotyping of F1534C mutation in adult *Ae. aegypti* from BUK. (**a**) ƛ-cyhalothrin-alive females, (**b**) ƛ-cyhalothrin-dead, (**c**) DDT-alive (1-9) and DDT-dead (9-15). L is hyperladder IV DNA ladder (Bioline 100–1013 bp). A band of 350 bp represent control band, while 231 bp is for wild-type (phenylalanine allele) and 167 bp is for *kdr* mutant (cysteine allele), with heterozygotes having both 231 bp and 167 bp bands.
