# Supplementary material for: Temporal Evaluation of Insecticide Resistance in Populations of the Major Arboviral Vector Aedes Aegypti from Northern Nigeria
Source: Insects. 2022 Feb 10;13(2):187. doi: 10.3390/insects13020187 (PMC8876019; doi:10.3390/insects13020187)
Supplement: Supplementary file 1 [file insects-13-00187-s001.zip › Figure S3.pdf]

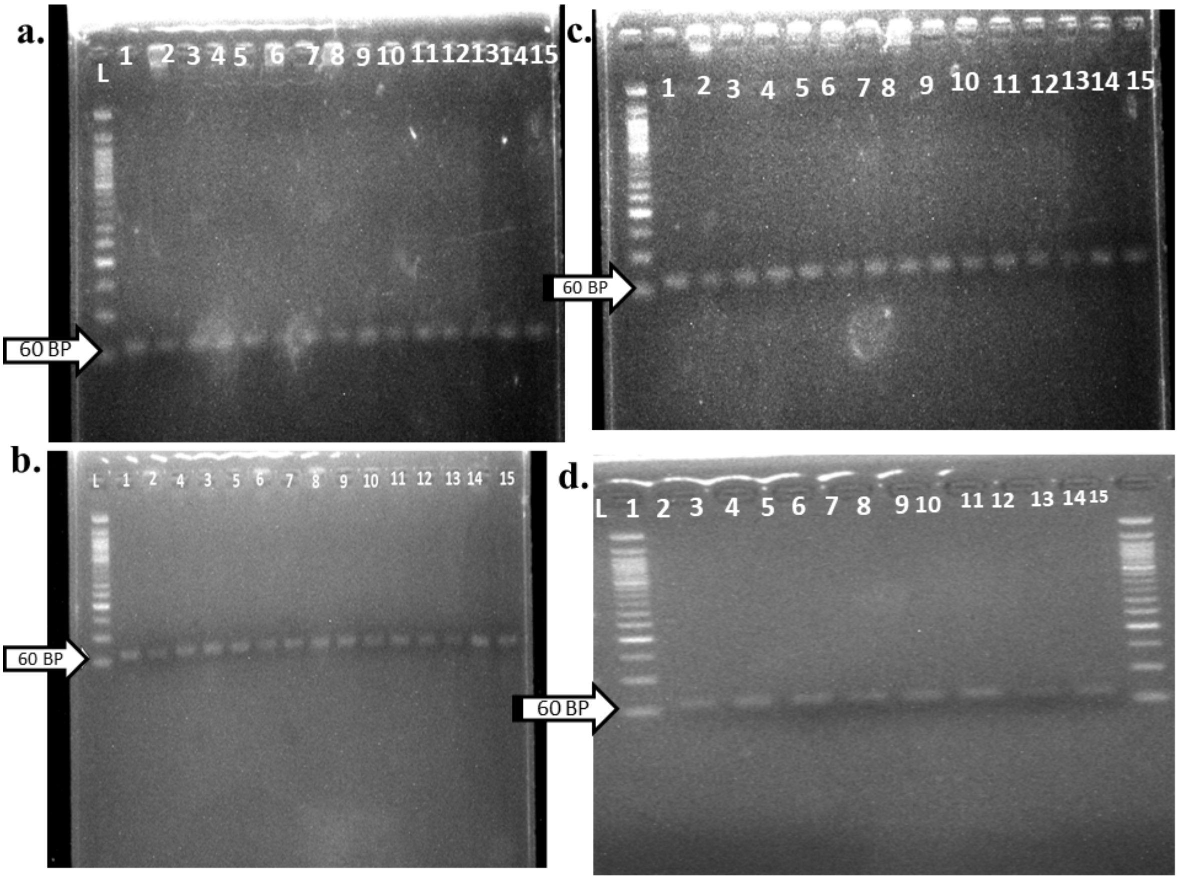

Figure S3. Agarose gel of allele specific PCR genotyping of V1016G mutation in female *Ae. aegypti* from BUK. (**a**,**b**) λ-cyhalothrin-alive, (**c**,**d**). λ-cyhalothrin-dead. L is 50 bp DNA ladder (NEB, 50–1350 bp). A band size of 80 bp fragment indicates homozygote resistant allele (GTA for valine) while 60 bp indicates homozygote susceptible allele (GGA for glycine).
